# Cosmeceutical activities of ethanol extract and its ethyl acetate fraction from coffee silverskin

**DOI:** 10.1186/s40824-018-0151-9

**Published:** 2019-01-14

**Authors:** Song Hua Xuan, Keon Soo Lee, Hyo Jin Jeong, Young Min Park, Ji Hoon Ha, Soo Nam Park

**Affiliations:** Department of Fine Chemistry, Cosmetic R&D center, Cosmetic Industry Coupled Collaboration Center, Seoul National University of Science and Technology, 232, Gongneung-ro, Nowon-gu, Seoul, 01811 Korea

**Keywords:** Coffee silverskin, Reactive oxygen species, Antioxidant activity, Cytoprotective effect, Metalloproteinase-1, Melanogenesis

## Abstract

**Background:**

Coffee silverskin is a thin film that covers the raw coffee bean. In general, coffee silverskin, which detaches during the coffee roasting process, is disposed as firelighters or dispatched to landfills and can cause serious environmental pollution. The aim of this study was to investigate the feasibility of using coffee silverskin as a functional material in cosmetics by evaluating its bioactive ingredients, antioxidative activity, cytoprotective effect, matrix metalloproteinase-1 (MMP-1)-inhibiting effect, and anti-melanogenesis effect.

**Results:**

To this end, a 50% ethanol (EtOH) extract and its ethyl acetate (EtOAc) fraction were prepared from coffee silverskin; caffeine was found to be the major compound in the extract. Both the 50% EtOH extract and its EtOAc fraction exhibited antioxidant activities. However, the EtOAc fraction showed a greater radical-scavenging activity and reducing power than that shown by the 50% EtOH extract. Furthermore, the EtOAc fraction increased cell viability in a UVB-irradiated human keratinocyte injury model and significantly suppressed UVB-induced MMP-1 expression and α-melanocyte-stimulating hormone (α-MSH)-stimulated melanin production in HaCaT keratinocytes and B16F1 melanocytes, respectively. Interestingly, caffeine, the major component of the EtOAc fraction, did not show an inhibitory effect. Thus, the antioxidant capacity of the coffee silverskin extract may be attributable to some compounds that exhibit a high antioxidant capacity even at low concentrations or the total antioxidant capacity of various constituent phenolic compounds.

**Conclusion:**

Our findings indicate that coffee silverskin has the potential for application as a natural functional material in multifunctional cosmetics.

## Background

The demand for functional cosmetics is on the rise because of population aging and an increasing interest in health and beauty. Furthermore, it has been long known that Asians, particularly the Korean, Chinese, and Japanese people, are interested in functional cosmetics, especially whitening products. In recent years, there has been an increasing trend of developing multifunctional cosmetics with benefits such as anti-aging, whitening, moisturizing, and ultraviolet (UV) radiation-protective efficacy. Sunlight exposure due to outdoor activities leads to ultraviolet light directly acting on the skin, the largest organ of the human body and the first protective shield against the external environment [1]. UV irradiation not only induces DNA damage by causing the formation of cyclobutane–pyrimidine dimers (CPDs) and pyrimidine-pyrimidone (6–4) photoproducts in cells but also causes the generation of a variety of reactive oxygen species (ROS) such as singlet oxygen (^1^O_2_), superoxide anion radical (O_2_^•−^), hydrogen peroxide (H_2_O_2_), hydroxyl radical (• OH), alkoxyl radical (• OR), and peroxyl radical (• OOR) [2]. UV-induced ROS accelerate skin aging by initiating protein oxidation and lipid peroxidation and promoting the expression of matrix metalloproteinases (MMPs) to breakdown collagen and elastin fibers, which are the matrix components of the dermis layer [3]. In addition to photoaging, facial spots and freckles are caused by the accumulation of oxidative stress through excessive melanin production [4, 5]. Therefore, it is essential to develop a multifunctional cosmetic with whitening and anti-photoaging effects, along with human skin cell-protective activity characterized by an effective removal of excess ROS due to UV radiation or absorption of UV radiation [6].

Coffee is the world’s most widely consumed beverage, with its consumption increasing every year [7]. As reported by the International Coffee Organization, coffee consumption increased with a compound annual growth rate of 1.9% from 148 million of 60 kg bags in 2013 to 157 million in 2017 [8]. Large amounts of residues are generated in the process of brewing coffee, which can cause serious environmental pollution. The thin film that covers the raw coffee bean is called coffee silverskin [9], which detaches during the coffee roasting process. Roasted coffee beans are ground and brewed with near-boiling water to prepare coffee; most of the silverskin is disposed as firelighters or for landfills [10]. Recently, however, coffee silverskin has been reported to have several biological activities such as prebiotic characteristics [11] and hyaluronidase inhibitory activity [12]. Because of its application value, interest in coffee silverskin has gradually increased. However, there is still not much evidence of its effect on total antioxidant activity in Fe^3+^-EDTA / H_2_O_2_ system, cytoprotective effect against UV-induced human keratinocyte damage, and anti-photoaging and whitening activity. Therefore, the present study was conducted to investigate the feasibility of using coffee silverskin as a functional cosmetic by evaluating its bioactive ingredients, antioxidative activity, cytoprotective effect, and inhibition of MMP-1 and melanin production activities.

## Methods

### Reagents and chemicals

Folin-Ciocalteu’s phenol reagent, the 2,2-diphenyl-1-picrylhydrazyl (DPPH) radical, luminol, ethylenediaminetetraacetic acid (EDTA), H_2_O_2_, 3-(4,5-dimethythiazol-2-yl)-2,5-di-phenyltetrazolium bromide (MTT), FeCl_3·_6H_2_O, and L-ascorbic acid were purchased from Sigma-Aldrich (St. Louis, USA). Various solvents such as ethanol (EtOH) and ethyl acetate (EtOAc) were of analytical grade. In addition, Dulbecco’s modified Eagle’s medium (DMEM), fetal bovine serum, penicillin-streptomycin, and trypsin used for cell culture were purchased from Capricorn Scientific (Ebsdorfergrund, Germany).

### Preparation of the EtOH extract and its EtOAc fraction from coffee silverskin

**Fig. 1:**
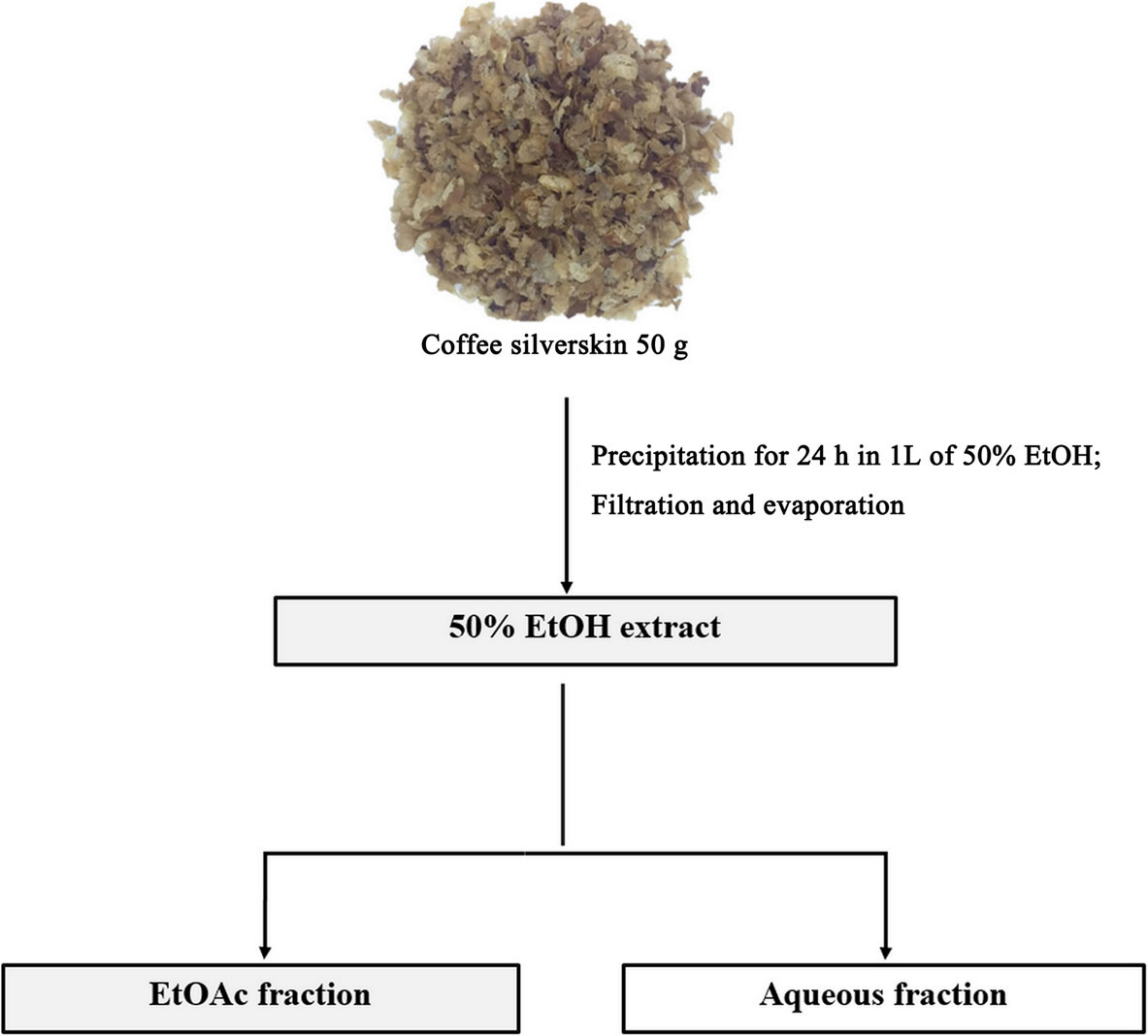
Fractionation scheme for the 50% EtOH extract of coffee silverskin and its EtOAc fraction

The coffee silverskin used in this study was obtained by roasting coffee beans (*Coffea arabica*) at the Roasting Barn Co. (Seoul, Korea). The process of preparing the 50% EtOH extract and its EtOAc fraction from coffee silverskin was as follows (Fig. 1). Dried coffee silverskin powder (50 g) was extracted with 1 L of 50% EtOH for 24 h at room temperature. The extracts were then filtered using a Buchner funnel and concentrated with a rotary evaporator to obtain a 50% EtOH extraction powder. The 50% EtOH extract was fractionated with EtOAc and concentrated with a rotary evaporator. Then, the dried samples were stored at − 80 °C in the refrigerator (Cosmetic R&D Center, Seoul National University of Science and Technology, Seoul, Korea) until use.

### Determination of total phenolic content

The total phenolic contents of the 50% EtOH extract of coffee silverskin and its EtOAc fraction were determined by slightly modifying the method described by Alves et al [13]. Briefly, 80 μL of the samples was added to 20 μL of Folin-Ciocalteu reagent (50% *v*/v in H_2_O), mixed thoroughly, and incubated at room temperature for 5 min. Then, 200 μL of sodium carbonate solution (2% *w*/*v* in H_2_O) was added, and the mixture was incubated at room temperature for 30 min. Absorbance was measured at 760 nm by using an ELISA reader. The total phenolic contents were determined by preparing a standard curve of concentration (0–50 mg/L) by using chlorogenic acid as a reference material (y = 0.0078 x + 0.0226, *R*^*2*^ = 0.9968).

### Characterization of coffee silverskin extracts by using HPLC and LC/ESI-MS

A component analysis of the silverskin extract and determination of caffeine content were performed using a Shimadzu LC-20A high-performance liquid chromatography (HPLC) system with a UVD 170 s DIONEX detector and Shim-pack VP-ODS C18 column (L: 250 mm, LD: 4.6 mm, 5 μm) (Shimadzu, Japan). The mobile phase was solvent A (2% acetic acid in H_2_O) and B (0.5% acetic acid in 50% acetonitrile aqueous solution), and the following linear scheme was used: 0–5 min, 0–10% (*v*/v) of B; 5–20 min, 10–100% (v/v) of B; 20–25 min, 100–100% (v/v) of B; 25–30 min, 100–10% (v/v) of B; and 30–35 min, 10–10% (v/v) of B. The flow rate was 1.0 mL/min, and the sample was confirmed at a wavelength of 254–400 nm. Quantitation of caffeine content was performed using a commercial standard (50–500 mg/L; *R*^*2*^ = 0.9955) at a maximum absorption wavelength of 270 nm. The mass spectrometric analysis was performed using an LCQ ion trap mass spectrometer (Thermo Finnigan) with an electrospray ionization (ESI) interface and detected in the positive ion mode by National Instrumentation Center for Environmental Management College of Seoul National University (Seoul, Korea). The operating conditions were as follows: capillary voltage, 33 V; capillary temperature, 400 °C; nebulizer pressure, 10 psi; and drying gas, N_2_.

### Free radical-scavenging activity

A free radical with an unpaired electron is unstable and very reactive. The high reactivity of free radicals damages not only the skin but also disturbs the body composition. To evaluate the free radical-scavenging activity of the EtOH extract of coffee silverskin and its EtOAc fraction, the reducing power of the sample was measured based on its electron-donating ability against DPPH, which is a relatively stable radical. An equal volume of EtOH and the sample (0.4 mL) was added to 0.4 mL of 0.2 mM DPPH solution dissolved in methanol. The samples were then mixed and allowed to stand at room temperature for 10 min. Absorbance was measured at 517 nm using a U*V*/Vis spectrophotometer [14]. (+)-α-Tocopherol was used as a positive control.

### ROS-scavenging activity using luminol-dependent chemiluminescence in the Fe^3+^-EDTA/H_2_O_2_ system

The Fe^3+^-EDTA/H_2_O_2_ system was used to determine the ROS-scavenging activity as described previously [14]. Various concentrations of the sample (50 μL) were added to 1.78 mL of distilled water and then 40 μL of EDTA (2.5 mM), 10 μL of FeCl_3·_6H_2_O (5 mM), and 80 μL of luminol (35 mM) were added. Then the tube was placed in the cell holder of the chemiluminescent device and incubated for 5 min. After incubation, 40 μL of H_2_O_2_ (150 mM) was added to the mixture, and chemiluminescence was measured for 25 min. In the control group, the solvent was added instead of the sample. The blank was the same as that used in the experiment but distilled water was added instead of H_2_O_2_ and FeCl_3_·6H_2_O. The ROS-scavenging activity (active oxygen-scavenging activity [OSC_50_]) was defined as the concentration of the sample required for 50% scavenging of ROS:

$$ \mathrm{ROS}\ \mathrm{Scavenging}\left(\%\right)=\frac{\ {\mathrm{cpm}}_{\mathrm{control}}-{\mathrm{cpm}}_{\mathrm{experiment}}}{{\mathrm{cpm}}_{\mathrm{control}}-{\mathrm{cpm}}_{\mathrm{blank}}}\times \mathrm{l}00 $$

### Measurement of intracellular ROS generation

Intracellular ROS generation was assessed using the fluorescence dye 2′,7′-dichlorodihydrofluorescein diacetate (H_2_DCF-DA, Sigma-Aldrich, USA) as our previous reports [3].

### Determination of the cytoprotective effect

HaCaT cells (CLS Cell Line Service, Germany) were cultured up to 70–80% confluence in a cell culture medium and then irradiated with 400 mJ/cm^2^ UVB (280–320 nm) in Dulbecco’s phosphate-buffered saline (DPBS) containing various doses of the samples by using a CL-1000 Ultraviolet Cross linker (UVP Co., USA) device. Our preliminary study showed that 400 mJ/cm^2^ of UVB irradiation reduced cell viability by about 70% compared with the unirradiated control (data not shown). After washing twice with DPBS, the cells were cultured for 24 h in serum-free DMEM culture conditions. The cytoprotective effect of the 50% EtOH extract of coffee silverskin and its EtOAc fraction against UVB-induced cell damage was measured by confirming cell viability by using the 3-(4,5-dimethyl-2-thiazolyl)-2,5-diphenyltetrazolium bromide (MTT; Sigma-Aldrich) assay as previously described [15].

### Quantitative analysis of MMP-1 expression

HaCaT cells were seeded into 60-mm plates and cultured up to 70–80% confluence in a cell incubator maintained at 37 °C. After treatment with different concentrations of the samples, the cells were exposed to 80 mJ/cm^2^ UVB, which does not affect cell survival, and then cultured further for 48 h in fresh medium. Immunoreactivity for MMP-1 within the culture medium was measured using an ELISA kit (R&D Systems, USA) according to the manufacturer’s instruction.

### Western blot analysis

Western blot analysis was performed as previously described [3] with specific antibodies against caspase-3 (Signalway Antibody, USA), MMP-1 (Abnova, Taiwan), and β-actin (Cell Signaling Technology, USA).

### Measurement of melanin content

B16F1 melanoma cells, which obtained from Kyung Hee University Skin Biotechnology Center (Seoul, Korea), were seeded in 6-well plates and cultured in a cell incubator until 70–80% confluence was achieved. Then the cells were treated with α-melanocyte-stimulating hormone (α-MSH) and various concentrations of the samples in a fresh medium and further incubated for 72 h. After washing with DPBS, the cells were collected and 100 μL of 1 N sodium hydroxide solution containing 10% DMSO was added to the pellet. Absorbance was measured at 410 nm using the ELISA microplate reader.

### Statistical analysis

The results were repeated at least three times and expressed as the mean ± standard deviation (S.D.) values. Statistical significance was determined by one-way ANOVA with SPSS 17.0 (SPSS Inc. Chicago, IL). Differences were considered statistically significant at *p <* 0.05.

## Results

### Yield and total phenolic compound content

As shown in Table 1, the yields of the 50% EtOH extract of coffee silverskin and its EtOAc fraction were 11.06% (*w*/w) and 0.91% (w/w), respectively, and were determined based on dried coffee silverskin (50 g). Phenolic compounds are natural antioxidants and known to be very important factors in determining the antioxidant activity of natural products [16]. In this study, the phenolic content was expressed as the amount contained in 1 g of the 50% EtOH extract or its EtOAc fraction or the amount contained in 1 g of dried coffee silverskin weight. The contents of phenolic compounds in the 50% EtOH extract of coffee silverskin and its EtOAc fraction were converted to the amount of chlorogenic acid, and the results show that the content in the EtOAc fraction (47.84 mg/g of coffee silverskin extract, 0.44 mg/g of coffee silverskin) was higher than that in the 50% EtOH extract (34.90 mg/g of coffee silverskin extract and 3.86 mg/g of coffee silverskin) (Table 1).

**Table 1:** Yields and Contents of Bioactive Compounds in the Coffee Silverskin 50% EtOH Extract and Its Fraction

Sample	Yields (%, g/g of dry weight)	Total phenolic content		Caffeine	
		mg/g of extract	mg/g of dry weight	mg/g of extract	mg/g of dry weight
50% EtOH extract	11.06	34.90 ± 4.90	3.86 ± 0.15	112.40 ± 0.21	12.43 ± 0.12
EtOAc fraction	0.91	47.84 ± 0.01	0.44 ± 0.31	388.29 ± 0.36	3.53 ± 0.06

Data are presented as mean ± S.D. values

### HPLC and LC/ESI-MS analyses and determination of the caffeine content in coffee silverskin

**Fig. 2:**
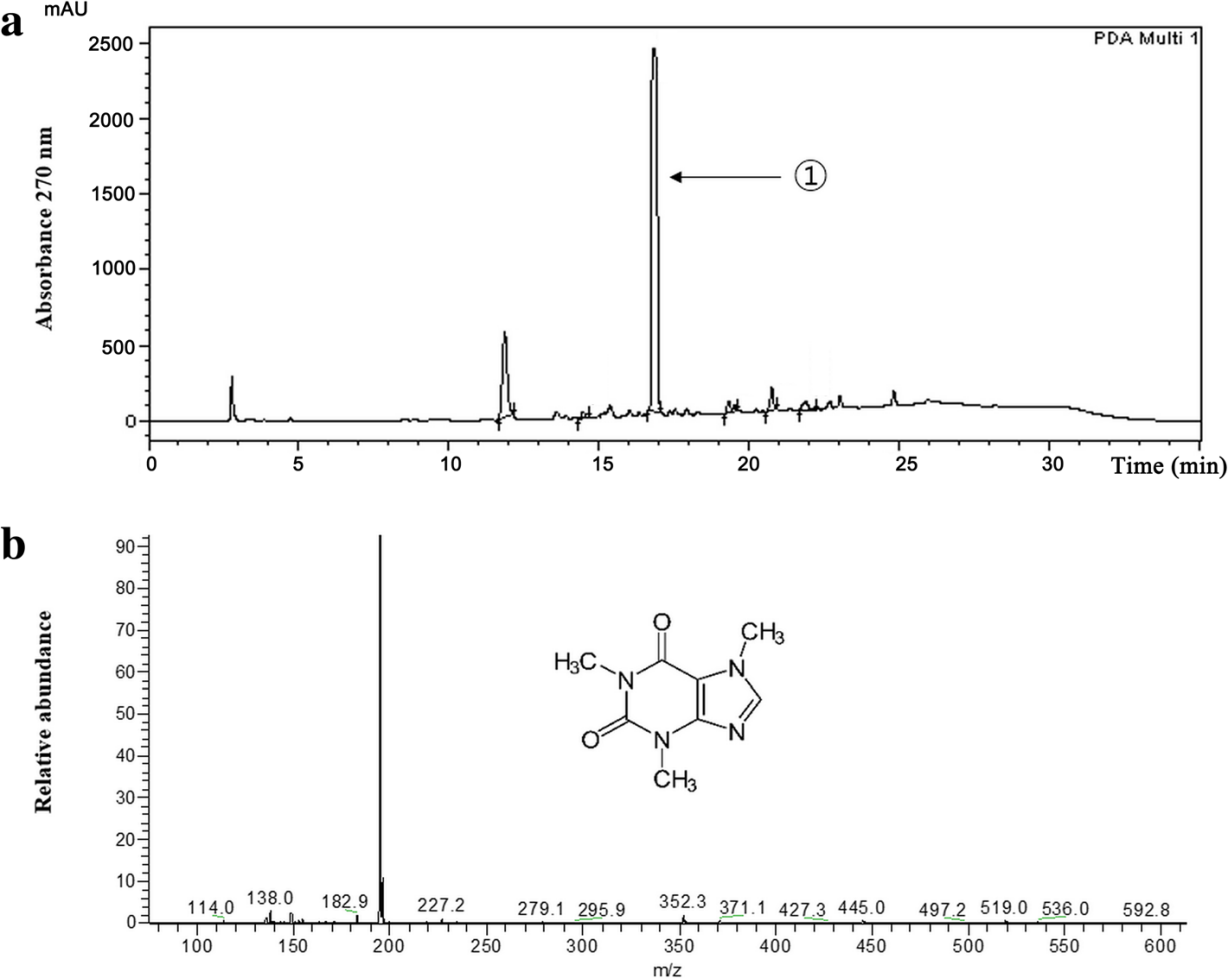
HPLC chromatogram of the EtOAc fraction of the coffee silverskin EtOH extract at 270 nm. ① Caffeine (**a**). Mass spectra of ① in positive mode (LC-MS) (**b**). Insert shows caffeine structure

HPLC and LC/ESI-MS were used to analyze the components of coffee silverskin. The HPLC analysis of the 50% EtOH extract of coffee silverskin and its EtOAc fraction showed that the retention time of the major components was 16.8 min and the retention times were consistent with those of caffeine standards (Fig. 2a). For a more accurate analysis, the [M-H]^+^ ion was detected at m/z 195 in the positive ion mode of LC/ESI-MS. Thus, the major component of the silverskin extract was identified as caffeine (Fig. 2b). Determination of the caffeine content using a standard calibration curve revealed the caffeine content in the 50% EtOH extract of coffee silverskin to be 112.40 mg/g and that in its EtOAc fraction to be 388.29 mg/g (Table 1).

### Free radical-scavenging activity

The free radical-scavenging activity of the coffee silverskin extracts was determined by DPPH assay, which is widely used for measuring antioxidant activity. In solution, the DPPH radical has a deep violet color, and it became pale yellow upon reaction with an antioxidant because of its antioxidative capacity [17]. Results showed that free radical scavenging activity (FSC_50_) values for the 50% EtOH extract and EtOAc fraction were 105.43 μg/mL and 65.27 μg/mL, respectively. However, caffeine, a major component of the coffee silverskin extract, did not show any free radical-scavenging activity and the FSC_50_ value of (+)-α-tocopherol was 8.98 μg/mL. These experiments indicated that the free radical-scavenging activity of the EtOAc fraction was higher than that of the 50% EtOH extract (Table 2). Although the FSC_50_ values were lower than that of (+)-α-tocopherol, which is known as a strong lipid-soluble antioxidant in the cell membrane, the radical-scavenging activity of both the 50% EtOH extract and EtOAc fraction was still significant.

**Table 2:** Antioxidant Activity of the 50% EtOH Extract of Coffee Silverskin and Its EtOAc Fraction and Reference

	Free radical-scavenging activity (FSC_50_, μg/mL)	ROS-scavenging activity (OSC_50_, μg/mL)
50% EtOH extract	105.43 ± 3.58	31.89 ± 31.94
EtOAc fraction	65.27 ± 3.32	15.25 ± 0.55
Caffeine	> 500	> 500
(+)-α-Tocopherol	8.98 ± 2.93	nd
L-Ascorbic acid	nd	1.50 ± 0.85

Data are presented as mean ± S.D. values

*nd* not detected

### ROS-scavenging activity

To study the total antioxidant activity of ROS scavengers, the Fe^3+^-EDTA/H_2_O_2_ system was used. In this system, most types of ROS are generated by Fenton reaction and subsequent chain reaction, including O_2_^•−^, H_2_O_2_, and • OH, except for ^1^O_2_. The resulting ROS are able to oxidize the luminol to form the aminophthalate in the excited state and emit luminescence (420–450 nm) [18]. Therefore, the luminol chemiluminescence assay can measure not only the reducing power of the samples but also the chelating activity of the inhibition of ROS generation. In this study, the total antioxidant capacity of the 50% EtOH extract of coffee silverskin and its EtOAc fraction, the water-soluble antioxidant L-ascorbic acid, and caffeine, which is the main component of the coffee silverskin extract, was determined, and the results are shown in terms of OSC_50_, which is the concentration at which active oxygen is reduced by 50%. It was found that the OSC_50_ of the coffee silverskin 50% EtOH extract was 31.89 μg/mL and that of the EtOAc fraction increased by about 50% to 15.25 μg/mL. However, both the 50% EtOH extract and the EtOAc fraction showed lower activity than that shown by L-ascorbic acid (1.50 μg/mL), which corresponded to the control group. Unsurprisingly, total antioxidant capacity was not observed for caffeine, the main component of coffee silverskin (Table 2).

### Inhibitory effect of coffee silverskin extract on intracellular ROS generation

**Fig. 3:**
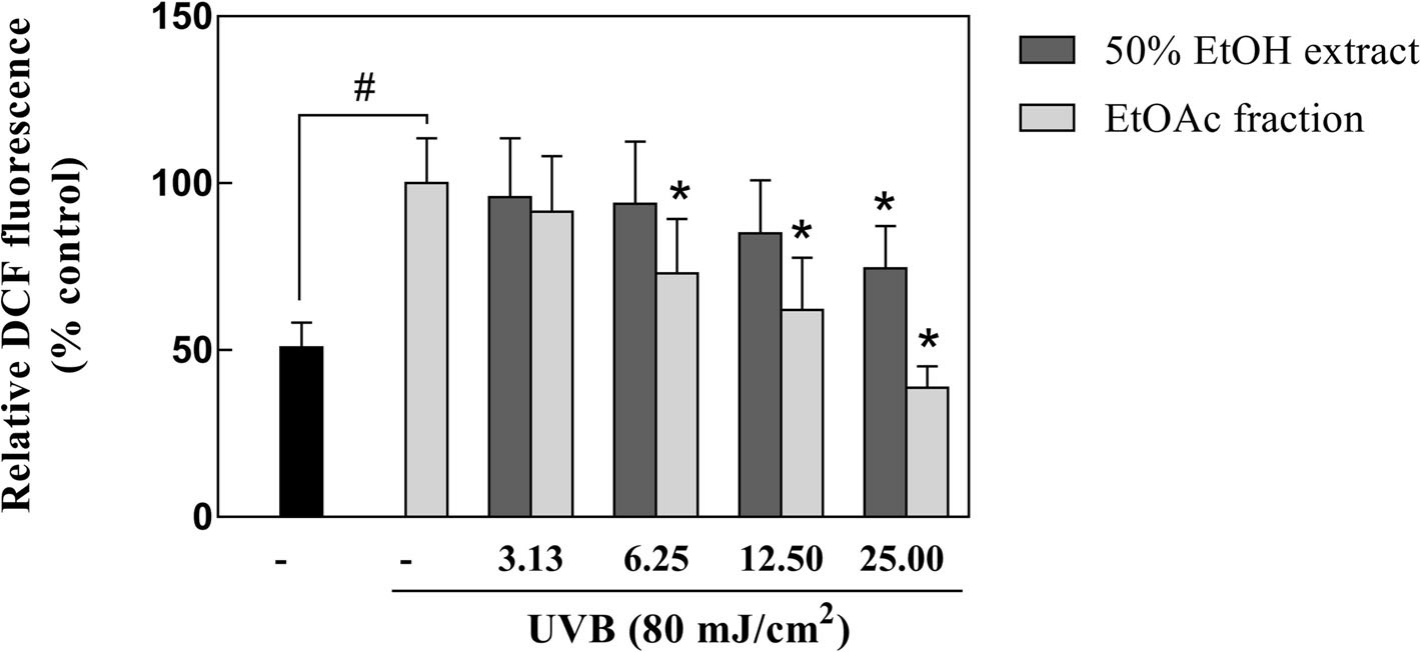
The effect of the 50% EtOH extract and EtOAc fraction of coffee silverskin on UVB-induced ROS generation in HaCaT cells. Data are presented as mean ± S.D. values. *p < 0.05 compared with the UVB-irradiated control, ^#^*p* < 0.05 compared with the UVB-non-irradiated control

To examine the effect of samples on intracellular ROS generation, HaCaT cells were treated with H_2_DCF-DA to measure the DFC content following UVB irradiation. The results showed that UVB irradiation significantly increased ROS production compared to that in the UVB-untreated control. In contrast, pretreatment with the EtOAc fraction of coffee silverskin drastically repressed the intracellular ROS level to 39% at concentrations of 25 μg/mL in comparison with the UVB irradiated cells. Although a decrease trend also observed in the 50% EtOH extract treated group, it had a lesser intracellular ROS inhibitory effect than that of the EtOAc fraction (Fig. 3).

### Cytoprotective effect of the coffee silverskin extract

**Fig. 4:**
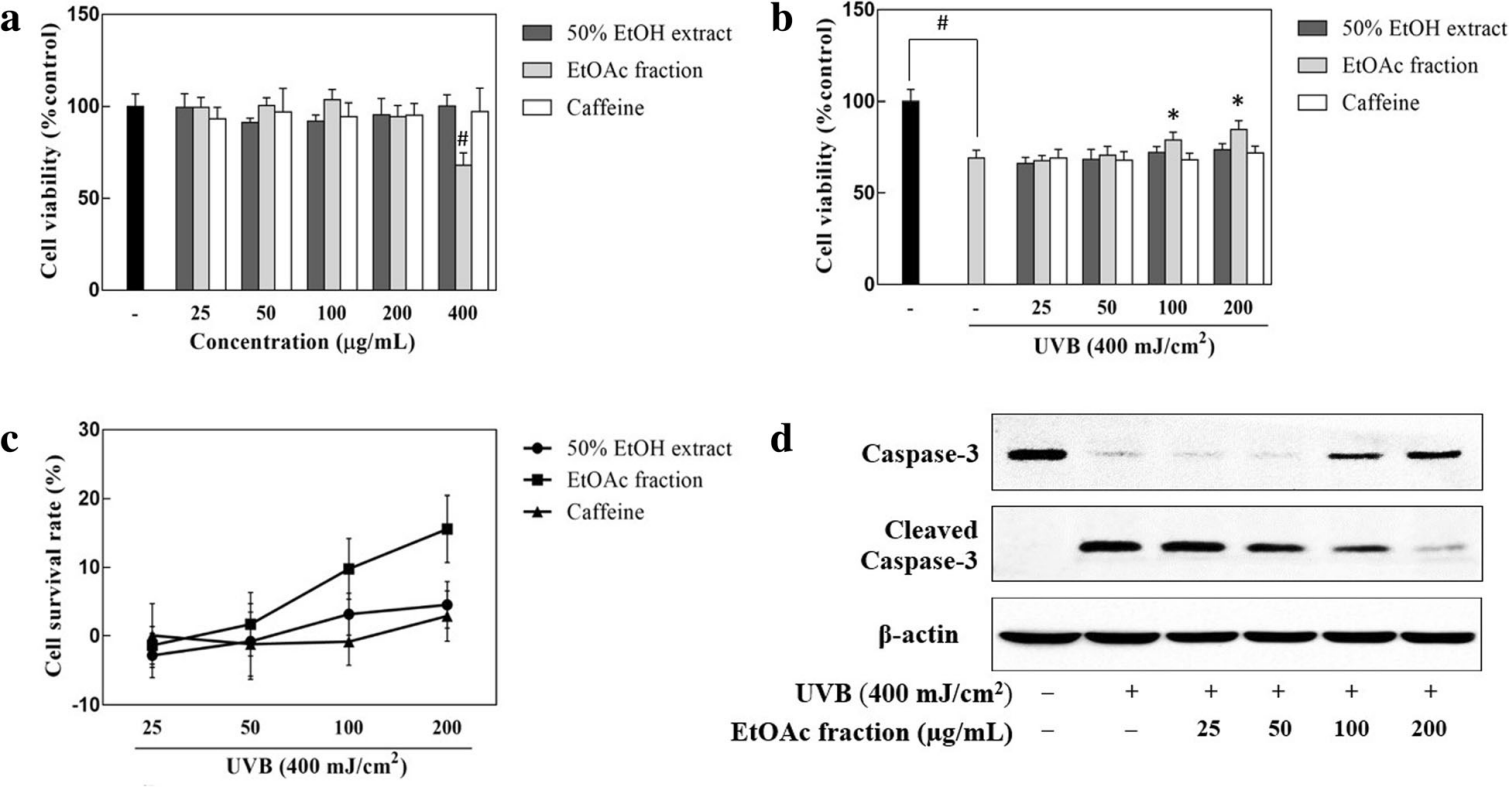
The effects of the 50% EtOH extract and EtOAc fraction of coffee silverskin in UVB-irradiated HaCaT cells. HaCaT cells were treated with different concentration of the samples in the absence (**a**) or presence (**b** and **c**) of 400 mJ/cm^2^ UVB. The cytotoxicity was then determined by the MTT assay 24 h after UVB irradiation. The effect of coffee silverskin EtOAc fraction on caspase-3 expression was detected by western blot analysis (**d**). Data are presented as mean ± S.D. values. *p < 0.05 compared with the UVB-irradiated control, ^#^*p* < 0.05 compared with the UVB-non-irradiated control. β-actin was used as an internal control

The concentration ranges of the samples used for these experiments were determined by confirming the cytotoxicity of the coffee silverskin extract and fractions, and caffeine in human keratinocytes by the MTT method. When 70–80% confluence was reached, the cells were treated with DPBS containing various doses of the samples during the 400 mJ/cm^2^ UVB irradiation. Treatment with the 50% EtOH extract and caffeine did not cause cytotoxicity at concentrations up to 400 μg/mL, and the EtOAc fraction had no significant cytotoxic effect at concentrations up to 200 μg/mL (Fig. 4a). Therefore, when measuring the cytoprotective effect of the samples, the cytoprotective concentration range of the samples was determined to be 25–200 μg/mL in the present experiment.

It is known that the sunburn-inducing ability of UVB is 1000-fold higher than that of UVA. The generated ROS were able to form abnormal covalent bonds such as those in pyrimidine dimer in skin cells and induce cell death by activation of the apoptotic pathway, with caspase-3 acting as an important regulator [19]. In this study, we analyzed whether the 50% EtOH extract and its EtOAc fraction possessed antioxidant activity against oxidative damage by UVB irradiation. As shown in Fig. 4, the experimental group irradiated with 400 mJ/cm^2^ UVB showed a survival rate of 69.16% compared to that of the UVB-untreated group, and the cell viability did not increase significantly at concentrations of 25–200 μg/mL in the 50% EtOH extract- and caffeine-treated groups. In contrast, significant increases in cell viability of 78.94 and 84.75% were observed compared to that in the irradiated controls when the cells were irradiated with 400 mJ/cm^2^ UVB in the presence of the EtOAc fraction of coffee silverskin at concentrations of 100 μg/mL and 200 μg/mL, respectively (Fig. 4b and c). We also observed that the expression level of caspase-3 decreased and a cleaved activated form has increased dramatically in HaCaT cells after exposure to 400 mJ/cm^2^ UVB, and the EtOAc fraction of coffee silverskin partially counteracted this effect (Fig. 4d). These data indicated that the EtOAc fraction of coffee silverskin showed a protective effect against UVB-induced cell damage via upregulation of caspase-3 expression in HaCaT cells.

### Inhibitory effect of coffee silverskin extract on UVB-induced MMP-1 expression

**Fig. 5:**
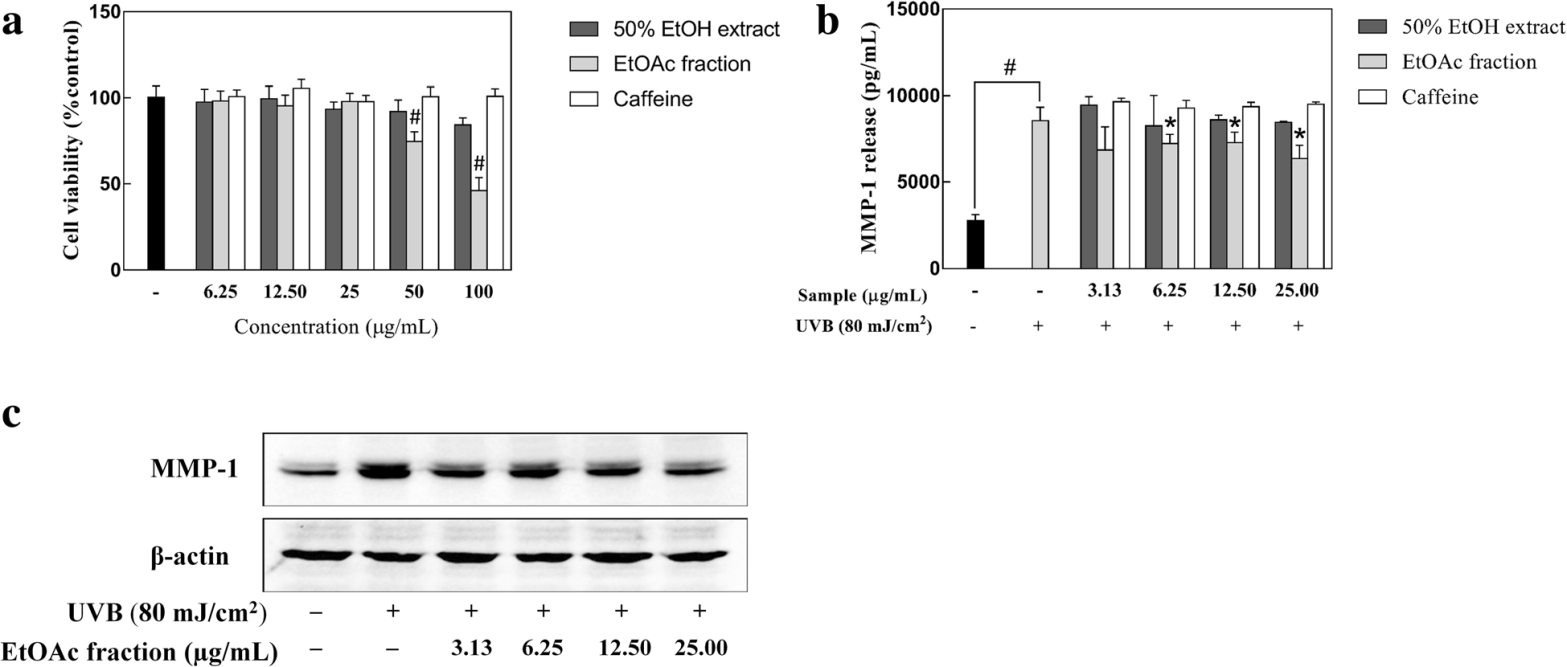
The effect of the 50% EtOH extract and EtOAc fraction of coffee silverskin on UVB-induced MMP-1 expression. Cell viability determined using the MTT assay (**a**). The levels of UVB-induced MMP-1 expression in HaCaT cells were measured by ELISA (**b**) and western blot analysis (**c**). Data are presented as the mean ± S.D. values. *p < 0.05 compared with the UVB-irradiated control, ^#^*p* < 0.05 compared with the unirradiated control. β-actin was used as an internal control

To determine the concentration range of the samples to be used in this experiment, the effects of the 50% EtOH extract, EtOAc fraction, and caffeine on the viability of HaCaT keratinocytes were evaluated by the MTT assay. When 70–80% confluence was reached, the cells were pretreated with various doses of the samples for 24 h and then cell viability was measured. Treatment with caffeine (6.25–400 μg/mL), and the 50% EtOH extract of coffee silverskin (6.25–50 μg/mL) and its EtOAc fraction (6.25–25.00 μg/mL) for 24 h had no cytotoxic effect on HaCaT keratinocytes (Fig. 5a). Therefore, when measuring the inhibitory effects of the samples on UVB-induced MMP-1 expression in HaCaT keratinocytes, the concentration range for the samples was determined to be 3.13–25.00 μg/mL in the present experiment.

ROS, which are generated excessively upon UV exposure, induce the expression of MMP-1, an enzyme that degrades the components of the extracellular matrix of the skin, and accelerate the aging process. The MMP-1 protein, which has a major role in the formation of wrinkles, is generated primarily in the skin cells, especially keratinocytes and fibroblasts [20, 21]. Therefore, in the present study, we investigated whether the 50% EtOH extract of coffee silverskin and its EtOAc fraction have antiaging effects against UVB-induced MMP-1 production in HaCaT keratinocytes. As shown in Fig. 5, in the experimental group irradiated with UVB, the expression level of MMP-1 increased by 310.90% compared to that in the UVB-untreated control. Treatment with the 50% EtOH extract and caffeine did not reduce the expression of MMP-1 significantly. However, a decreasing trend in MMP-1 release was observed as the concentration of the EtOAc fraction of coffee silverskin increased, and a significant decrease of 74.27% was observed compared to that in the UVB-treated control when the cells were pretreated with 25 μg/mL EtOAc fraction (Fig. 5b). Furthermore, western blot also presented a similar result that treatment of EtOAc fraction of coffee silverskin to HaCaT cells noticeably decreased the expression of MMP-1 (Fig. 5c). Collectively, these results showed that the EtOAc fraction of coffee silverskin had an inhibitory effect on UVB-induced MMP-1 expression.

### Inhibitory effect of coffee silverskin extract on melanin synthesis

**Fig. 6:**
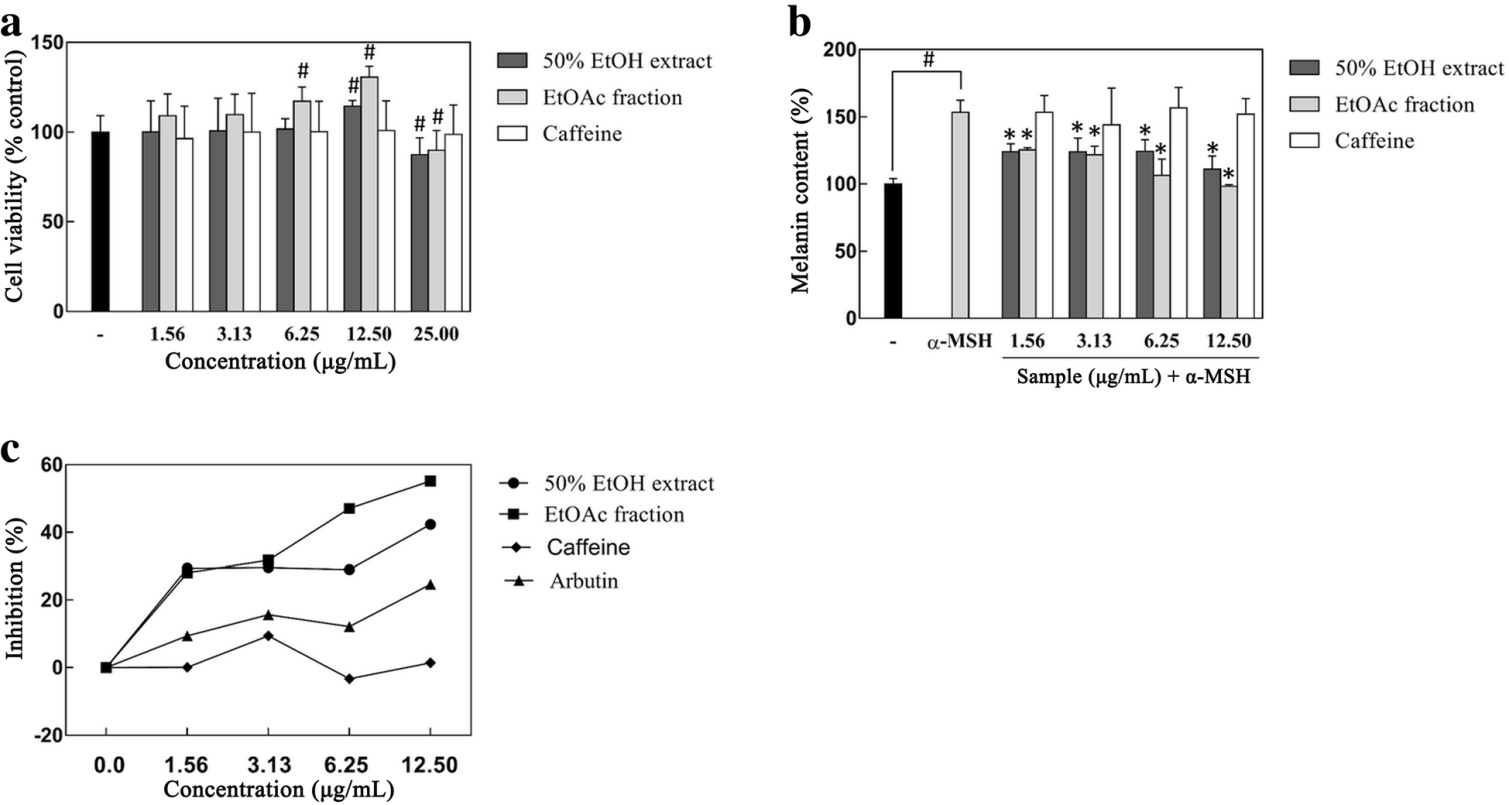
The effect of the 50% EtOH extract and EtOAc fraction of coffee silverskin on melanin synthesis. Cell viability determined using the MTT assay (**a**), effects of the 50% EtOH extract of coffee silverskin and its EtOAc fraction and caffeine on α-MSH-induced melanin synthesis (**b** and **c**) in B16F1 melanoma cells treated with different concentrations of the extract for 72 h. Data are presented as the mean ± S.D. values. *p < 0.05 compared with the α-MSH-treated control, ^#^*p* < 0.05 compared with the untreated control

To confirm the cytotoxicity of the samples in B16F1 melanoma cells, the MTT assay was used. The 50% EtOH extract and its EtOAc fraction did not show any cytotoxicity in the concentration range of 1.56 to 12.5 μg/mL, rather they increased cells viability significantly at the concentration of 12.5 μg/mL. Further, at the concentration of 25 μg/mL, cell viability markedly decreased compared to that in the untreated control (Fig. 6a). Therefore, the maximum concentration was set to 12.5 μg/mL when measuring the melanogenesis-inhibiting activity of the 50% EtOH extract of coffee silverskin and its EtOAc fraction.

α-MSH, which is induced by ultraviolet irradiation, is able to stimulate human melanocytes to accumulate excessive melanin; it also leads to the formation of dark spots and freckles, which affects people’s quality of life [22]. Therefore, we investigated the ability of the coffee silverskin extract to modulate melanogenesis in α-MSH-stimulated B16F1 melanoma cell model. Figure 5b shows that in the cells treated with α-MSH, the production of melanin increased about 1.4 times compared to that in the α-MSH-untreated control. In contrast, melanin synthesis decreased in groups treated with both the 50% EtOH extract of coffee silverskin and its EtOAc fraction. Combined with the above results of cell viability, both the 50% EtOH extract of coffee silverskin and its EtOAc fraction did not affect the B16F1 cell viability in the concentration range of 1.56 to 12.5 μg/mL, demonstrating that the suppression of melanin synthesis on this cells are not due to the cytotoxic effect of samples. In particular, the EtOAc fraction had a greater melanin biosynthesis inhibitory effect than that of the 50% EtOH extract. However, no statistically significant decreasing trend was observed for melanin contents in the B16F1 melanoma cells pretreated with different caffeine concentrations, indicating that the melanogenesis inhibitory effect of the coffee silverskin extract does not depend on caffeine (Fig. 6b and c).

## Discussion

UV radiation from the sun is classified as UVA (320–400 nm), UVB (280–320 nm), and UVC (200–280 nm) according to its wavelength. UVA (95–98% of the total UV radiation) and UVB (2–5% of the total UV radiation) reach the surface of the earth, thereby affecting the human body, unlike UVC, which is blocked by the ozone layer [23]. The biological impact of UVB exposure on the skin is much greater than that of UVA, at the same dose. UVB not only directly damages the structure of human skin cells but also leads to ROS generation, thereby affecting other biomolecules such as lipids and proteins, and the photodamage caused as a result is an important contributor to the degradation of extracellular matrix components and accumulation of excessive melanin [24]. However, these events can be reduced by treatment with antioxidants, such as phenolic compounds [25, 26]. Therefore, in this study, a 50% EtOH extract and EtOAc fraction of coffee silverskin, which is treated as waste in everyday life and causes environmental pollution, were prepared and analyzed for their antioxidant, cytoprotective, MMP-1-inhibiting, and melanogenesis-inhibiting effects to explore the possibility of using coffee silverskin as a multifunctional cosmetic material.

The antioxidative activities of the 50% EtOH extract of coffee silverskin and its EtOAc fraction were evaluated by determining the FSC_50_, total antioxidant ability (OSC_50_), and intracellular ROS levels. The FSC_50_ of the 50% EtOH extract and EtOAc fraction was 105.43 and 65.27 μg/mL, respectively, and DPPH assay revealed that the EtOAc fraction had higher radical-scavenging activity than that shown by the 50% EtOH extract. In the Fe^3+^-EDTA/H_2_O_2_ system using luminol, the total antioxidant activity of the silverskin EtOAc fraction (15.25 μg/mL) was also higher than that of the 50% EtOH extract (31.89 μg/mL). Although the EtOAc fraction had lower antioxidative activity than that of (+)-α-tocopherol, which is a strong lipid-soluble antioxidant, and L-ascorbic acid, a water-soluble antioxidant, it still showed a significant level of antioxidant activity. In addition, measurement of intracellular ROS levels also found that the silverskin EtOAc fraction was also higher than that of the 50% EtOH extract. The antioxidative effect of caffeine was more than 500 μg/mL in terms of both FSC_50_ and OSC_50_. Therefore, the antioxidative effect of the coffee silverskin EtOAc fraction is not attributable to caffeine, which was the major component of the coffee silverskin extract. In addition, the total phenolic content of the EtOAc fraction was 47.84 ± 0.01 mg/g, as determined by a modified Folin-Ciocalteu assay. Therefore, the antioxidant capacity of the EtOAc fraction is not determined by caffeine, but by some compounds that exhibit a high antioxidant capacity even at low concentrations or the total antioxidant capacity of various phenolic compounds.

UVB radiation has been reported to cause the formation of abnormal covalent bonds such as those in pyrimidine dimers in skin cells and induce DNA damage, lipid peroxidation, and skin aging. Our results using the UVB-induced human keratinocyte injury model indicated that the 50% EtOH extract of coffee silverskin and caffeine did not increase the UVB-induced cell death; however, the cell viability significantly increased in the EtOAc fraction-treated group. The absorption spectra of the EtOAc fraction were determined to confirm the possible causes that may account for the cytoprotective effect of the coffee silverskin EtOAc fraction on human keratinocytes exposed to UVB (data not shown). As a result, the maximum absorption wavelength of the EtOAc fraction was approximately 275 nm within the UVB spectra, which was consistent with the findings obtained for pure caffeine. However, treatment with pure caffeine at the same concentration as that used for treatment with the EtOAc fraction did not show a cytoprotective effect. The antioxidant test results showed that the coffee silverskin EtOAc fraction had a considerable antioxidant activity. Thus, the absorption effect of the EtOAc fraction at the wavelength tested might not contribute to its cytoprotective capacity, and the fraction likely has an indirect protective effect by eliminating UVB-induced ROS production. Moreover, our results showed that the EtOAc fraction of coffee silverskin partially decrease the activation levels of caspase-3 in UVB-irradiated HaCaT cells. All of these findings demonstrate that the EtOAc fraction of coffee silverskin against UVB-induced cell damage by decrease of reactive oxygen species and caspase-3 activation.

ROS, which produced upon UVB exposure, can increase MMP-1 expression in keratinocytes by activating the mitogen-activated protein kinase pathway and stimulating the activator protein-1 (AP-1) transcription factor. An increase in MMP-1 expression has been shown to cause degradation of collagen I, II, III, VII, and X type fibers, and affect skin aging [27]. Additionally, ROS can induce α-MSH and promote melanogenesis in melanocytes. Melanin is a major determinant of skin and hair color, and it is synthesized by melanocytes, which are present in the epidermal basal layer. The presence of melanin in an appropriate amount inhibits UV radiation-induced damage to skin cells, but excessive melanin production causes pigmentation such as freckles and facial spots [28, 29]. In the present study, it was confirmed that the EtOAc fraction of the 50% EtOH extract inhibits both UVB-induced MMP-1 expression and α-MSH-induced melanin production. However, caffeine, a major component of the EtOAc fraction, showed no inhibitory effect on MMP-1 expression and melanin production. Additionally, recent studies have shown that the main phenolic compounds in the coffee silverskin extract are caffeoylquinic acid lactone, 3-coumaroylquinic acid, 5-coumaroylquinic acid, 3-caffeoylquinic acid, 5-caffeoylquinic acid, 3,5-dicaffeoylquinic acid, and feruloylquinic acid [9, 30]. Hence, the inhibitory effect of the coffee silverskin EtOAc fraction on MMP-1 expression and melanin production is not attributable to caffeine, but perhaps to the total activity of various phenolic compounds.

## Conclusions

In conclusion, the present study demonstrated that coffee silverskin has the potential for application as a natural functional material in multifunctional cosmetics, because of its antioxidant activity, cytoprotective capacity, inhibition of MMP-1 expression, and anti-melanogenesis activity.
